# Expression of Kir4.1 and Kir5.1 inwardly rectifying potassium channels in oligodendrocytes, the myelinating cells of the CNS

**DOI:** 10.1007/s00429-016-1199-8

**Published:** 2016-02-15

**Authors:** C. Brasko, V. Hawkins, I. Chacon De La Rocha, A. M. Butt

**Affiliations:** Institute of Biology and Biomedical Sciences, School of Pharmacy and Biomedical Sciences, University of Portsmouth, St Michael’s Building, White Swan Road, Portsmouth, PO1 2DT UK

**Keywords:** Inward rectifying potassium channel, Glia, Astrocyte, Oligodendrocyte, White matter, Potassium regulation

## Abstract

The inwardly rectifying K^+^ channel subtype Kir5.1 is only functional as a heteromeric channel with Kir4.1. In the CNS, Kir4.1 is localised to astrocytes and is the molecular basis of their strongly negative membrane potential. Oligodendrocytes are the specialised myelinating glia of the CNS and their resting membrane potential provides the driving force for ion and water transport that is essential for myelination. However, little is known about the ion channel profile of mature myelinating oligodendrocytes. Here, we identify for the first time colocalization of Kir5.1 with Kir4.1 in oligodendrocytes in white matter. Immunolocalization with membrane-bound Na^+^/K^+^-ATPase and western blot of the plasma membrane fraction of the optic nerve, a typical CNS white matter tract containing axons and the oligodendrocytes that myelinate them, demonstrates that Kir4.1 and Kir5.1 are colocalized on oligodendrocyte cell membranes. Co-immunoprecipitation provides evidence that oligodendrocytes and astrocytes express a combination of homomeric Kir4.1 and heteromeric Kir4.1/Kir5.1 channels. Genetic knock-out and shRNA to ablate Kir4.1 indicates plasmalemmal expression of Kir5.1 in glia is largely dependent on Kir4.1 and the plasmalemmal anchoring protein PSD-95. The results demonstrate that, in addition to astrocytes, oligodendrocytes express both homomeric Kir4.1 and heteromeric Kir4.1/Kir5.1 channels. In astrocytes, these channels are essential to their key functions of K^+^ uptake and CO_2_/H^+^ chemosensation. We propose Kir4.1/Kir5.1 channels have equivalent functions in oligodendrocytes, maintaining myelin integrity in the face of large ionic shifts associated with action potential propagation along myelinated axons.

## Introduction

Glial cell function is largely determined by their expression of a wide range of plasmalemmal potassium channels (Verkhratsky and Steinhäuser 2000). The inwardly rectifying potassium channel subtype Kir4.1 is the dominant K^+^ channel in astrocytes (Kofuji and Newman 2004). Studies using the global Kir4.1 knock-out and targeted ablation have demonstrated the critical role for Kir4.1 in the key astroglial function of potassium homeostasis (Kofuji et al. 2000; Neusch et al. 2006; Djukic et al. 2007; Seifert et al. 2009; Bay and Butt 2013). In addition, astrocytes express multiple Kir subtypes and their functional diversity is increased by the formation of both homomeric and heteromeric channels (Hibino et al. 2010; Butt and Kalsi 2006). Kir5.1 is an inwardly rectifying K^+^ channel subunit that forms functional heteromeric channels with Kir4.1 (Konstas et al. 2003). Heteromeric Kir4.1/5.1 channels are highly pH sensitive and have biophysical properties distinct from homomeric Kir4.1 channels (Tanemoto et al. 2000; Pessia et al. 2001). Heteromeric Kir4.1/Kir5.1 channels have been indicated in astrocytes of the retrotrapezoid nucleus (RTN), where due to their special pH/CO_2_ sensitivity they are important in chemoreception and contribute to respiratory drive (Mulkey and Wenker 2011). The relationship between expression of Kir5.1 with the dominant astrocyte channel Kir4.1 is not fully elucidated, but it is possible that homomeric Kir4.1 and heteromeric Kir5.1/Kir4.1 channels may be differentially expressed in specific astroglial populations (Ishii et al. 2003; Hibino et al. 2004).

The other main class of glia in the CNS are oligodendrocytes, which have the essential function of myelinating axons that make up CNS white matter. In contrast to astrocytes, the ion channel profile of mature myelinating oligodendrocytes is unresolved. There is immunohistochemical evidence that oligodendrocytes express Kir4.1 (Poopalasundaram et al. 2000; Kalsi et al. 2004), and a study in the global Kir4.1 knock-out mouse demonstrates it is essential for oligodendrocyte development and myelination (Neusch et al. 2001). However, oligodendroglial expression of Kir4.1 is controversial (Hibino et al. 2004; Tang et al. 2009), and Kir5.1 has not been studied previously in an oligodendroglial context. Here, we have determined the expression of Kir5.1 and Kir4.1 in oligodendrocytes in the white matter of the mouse cerebellum and optic nerve, in comparison to astrocytes. The optic nerve is a typical CNS white matter tract that contains the axons of retinal ganglion cells and the glia that support them and is ideal for examining myelinating oligodendrocytes (Butt et al. 2004). The results demonstrate that oligodendrocytes, like astrocytes, express at least two subsets of Kir channels, homomeric Kir4.1 channels and heteromeric Kir4.1/5.1 channels. Oligodendroglial K^+^ channels are essential for myelin formation and integrity in the face of depolarizing elements (Hawkins and Butt 2013; Neusch et al. 2001; Menichella et al. 2006). The special biophysical properties of homomeric Kir4.1 and heteromeric Kir4.1/Kir5.1 channels indicate they have distinct physiological roles in maintaining oligodendrocyte function during the large shifts in K^+^ and pH to which they are exposed during action potential propagation.

## Materials and methods

### Experimental animals

All animals were killed by cervical dislocation, in accordance with regulations issued by the Home Office of the United Kingdom under the Animals (Scientific Procedures) Act, 1986. The animals used were C57BL6/J wild type strains and GFAP-EGFP, PLP-DsRed, Sox10-EGFP and Kir4.1 knock out transgenic mouse strains: GFAP-EGFP mice express enhanced green fluorescent protein (EGFP) under the control of the astrocyte-specific glial fibrillary acidic protein (GFAP) promoter (Nolte et al. 2001); PLP-DsRed mice express DsRed under the control of the oligodendrocyte-specific proteolipid protein (PLP) promoter (Hirrlinger et al. 2005); Sox10-EGFP mice express EGFP under the control of the Sox10 promoter, which in the CNS is specific to oligodendrocyte lineage cells (Stolt et al. 2006); the GFAP-EGFP and PLP-DsRed strains were kindly provided by Frank Kirchhoff (Molecular Physiology, University of Saarland, Homburg, Germany) and the Sox10-EGFP by Bill Richardson (Wolfson Institute for Biomedical Research, University College London, UK). The K_ir_4.1 channel knock-out mice were originally generated by Kofuji et al. (2000), and were kindly provided by Christian Steinhäuser (Institute of Cellular Neurosciences, University of Bonn, Germany). Kir4.1 KO mice were kept in a heterozygous background and genotyping was performed to identify homozygous, heterozygous and wild type offspring using the Mouse Tail Quick Extraction Solutions (Biopioneer Inc) and PCR amplification, as previously described (Kofuji et al. 2000).

### Optic nerve explant cultures

Optic nerve explant cultures were prepared from mice aged postnatal day (P)7–11, as described previously (Hawkins and Butt 2013). In brief, optic nerves were carefully dissected and maintained in pre-warmed (37 °C) and pre-gassed (95 % O_2_/5 % CO_2_) dissecting media, constituted of Dulbecco’s Modified Eagle Medium (DMEM) (Sigma) medium, supplemented with 4 mM l-glutamate, 10 % foetal bovine serum (FBS; Invitrogen) and 0.1 % gentamicin (Invitrogen, Life Technologies Ltd., Paisley). From this point on optic nerves were kept under sterile conditions and cut into 1–2 mm fragments in filter sterilized pre-warmed dissecting media, using a scalpel blade. For further dissociation, optic nerve fragments were triturated and nerve fragments transferred onto laminin (Invitrogen) and poly-l-ysine (Sigma) coated coverslips and incubated in dissecting medium at 37 °C in 95 % O_2_/5 % CO_2_ overnight to allow the adhesion of the explants. After 24 h, the dissecting media was substituted with a low serum (0.5 %) modified Bottenstein and Sato (B&S) culture medium, supplemented with 10 ng/ml recombinant human PDGF-AA (R&D Systems) and 0.1 % gentamicin. After 3–4 days in vitro (DIV) the medium was replaced to B&S media supplemented with 0.5 mM dibutyryl cAMP for up to 10 DIV. Explanted cells were then cultured in B&S media with 0.1 % gentamicin for up to 15 DIV, changing media every 3–5 days.

### Transfection

Optic nerve explant cultures were transfected at 10 DIV with psiSTRIKE vectors containing GFP and either Kir4.1 siRNA (forward 5′-ACCGCTCTTCTCTGCAACCTTTAAGTTCTCTAAAGGTTGCAGA-GAAGAGCTTTTTC-3′, reverse 5′-TGCAGAAAAAGCTCTTCTCTGCAACCTTTAGAG-AACTTAAAGGTTGCAGAGAAGAG-3′), or for scrambled control (forward 5′-ACCGACCTTCCTCCTTTT-AGTAAGTTCTCTACG-TAAAAGGAGGAAGGTCTTTTTC-3′, reverse 5′-TGCAGAAA-AAGACCTTCCTCCTTTTACGTAGAGAACTTACGTAAAAGGAGGAAGGT-3), using Lipofectamine™ 2000 reagent (Invitrogen) according to the manufacturer’s instructions. Cells were incubated at 37 °C in 95 % O_2_/5 % CO_2_ and examined at 2 days post transfection and transfected cells were identified by expression of GFP.

### Immunohistochemistry

Brain tissue and optic nerve explant cultures were fixed in 1 % paraformaldehyde in phosphate buffered saline (PBS, pH 7.4); tissue was fixed for either 1 h at RT or overnight at 4 °C, and for 10 min for cultured cells on their coverslips, followed by washes in PBS. For sectioning, brain tissues were placed in cryoprotectant (30 % w v^−1^ sucrose in PBS) overnight at 4 °C, then embedded in Cryo-M-Bed (Bright Instruments Company Ltd.), before rapidly freezing at −70 °C, sectioning using a cryostat (10 μm; Leica CM3050 S), and transference of sections onto Polysine^®^ coated slides (Thermo-Scientific). A blocking stage was performed by incubation in 5 % normal goat serum (NGS) for 1 h at RT, followed by permeabilization in 5 % NGS plus 0.2 % triton-X-100 in PBS. After blocking, samples were incubated overnight at 4 °C with primary antibodies diluted in NGS-PBS: chicken anti-GFAP, 1:500 (Chemicon); mouse anti-APC (adenomatous polyposis coli) antibody [CC-1], 1:700 (Calbiochem); rabbit anti-Olig2, 1:700 (Millipore); rabbit anti-Kir4.1, 1:400 (Kalsi et al. 2004); guinea-pig anti-Kir4.1, 1:300 (Alomone); rabbit anti-Kir4.1, 1:300 (Alomone); rabbit anti-Kir5.1, 1:300 (Alomone); goat anti-Kir5.1, 1:100 (Santa Cruz); mouse anti-Na/K ATPase α1, 1:300 (Abcam); mouse anti-PSD-95, 1:300 (Thermo). After washes in PBS, tissues and cells were incubated for 1 h at RT with the appropriate secondary antibodies: goat anti-rabbit ^488^Alexafluor, 1:400 (Molecular Probes); donkey anti-goat ^488^Alexafluor, 1:400 (Molecular Probes); goat anti-guinea-pig ^568^Alexafluor, 1:500 (Life Technologies); donkey anti-chicken ^568^Alexafluor, 1:400 (Molecular Probes); donkey anti-goat ^647^Dylight, 1:200 (Stratech); donkey anti-chicken ^405^Dylight, 1:400 (Stratech); donkey anti-rabbit ^647^Dylight, 1:200 (Stratech); donkey anti-mouse ^647^Dylight, 1:200 (Stratech). Hoechst Blue (0.2 μg/ml; Molecular Probes) was used to identify nuclei. Control experiments were carried out in which sections/cells were preabsorbed with antigen peptide overnight prior to incubation in the primary antibody, or where antigen peptide was not available controls were carried out by omitting primary antibody, and Kir4.1 antibodies were tested in sections/cells from Kir4.1 KO mice. Following immunolabeling, coverslips/sections were mounted with Vectasheild^®^ (VectorLabs) and images acquired using a Zeiss meta LSM 710 confocal microscopes (Zeiss), maintaining variables constant between images (see below).

### Image capture and analysis

Fluorescence was detected using excitation wavelengths of 488 nm (green), 568 nm (red), 633 nm (far red) and 405 nm (blue), with an argon, HeNe1 and diode laser, respectively. Confocal images were captured using the 40× or 63× oil immersion objectives (numerical apertures of 1.3 and 1.4, respectively), and images acquired using multi-track sequential capture, with optimal detector gain and offset acquisition settings for pinhole diameter 0.13–0.3 airy units, with an average of four scans per image, to detect positive signal with minimal background and prevent cross-talk between channels. Identical settings were used to image negative controls. *Z*-stacks were captured of 4–15 *z*-sections (voxel size 43–76 nm *x*–*y*, 76–283 nm *z*). Image analysis was carried out using Volocity 6.1 software (Improvision Ltd.). For confocal photomicrographs, two-dimensional flattened images of the *z*-stacks are presented. For colocalization analyses, the technique of Barlow and colleagues was used (Barlow et al. 2010), as previously described (Hawkins and Butt 2013), in which the degree of separation between pixels from the red and green channels was determined in single *z*-sections to provide measurements of signal overlap. First, images were thresholded to separate the positive signal (positive immunolabelling) from background; threshold was determined by measuring the background intensity value for each channel in negative control sections and setting the threshold as the mean background intensity plus three standard deviations (averaged from a minimum of 6 images). The thresholded Pearson’s correlation coefficient (PCC) and Mandersons’ overlap coefficients (M1 and M2) were determined as previously described (Barlow et al. 2010), using Volocity 6.1 software; thresholded PCC determines the statistical strength of the linear relationship between fluorescent intensities from the red and green channels, and the M1 and M2 overlap coefficients provide accurate measurements of the true degree of overlap of red and green. A colocalization channel was generated from the thresholded PCC to illustrate in three-dimensions the voxels in which the two channels overlap with the same intensity.

### Western blot

Whole brains or optic nerves were dissected from P16–P40 mice and homogenised in buffer containing: 12.5 mM NaCl; pH ~8 2 mM Tris/HCl; 0.2 mM phenyl-methyl sulphonyl fluoride (PMSF), distilled water and 1× complete mini protease inhibitor cocktail (Roche), all kept on ice. Samples were centrifuged at 12,000 rpm for 5 min at 4 °C and the aspirated supernatant was placed in a fresh tube on ice. Quantification of protein concentration was carried out with bicinchoninic acid assay (Sigma) with a standard bovine serum albumin (BSA) concentration curve and UV spectrophotometer absorbance readings at 550 nm. Samples were mixed with Laemmli sample buffer and heated at 95 °C for 5 min with β-mercaptoethanol (except samples used for the identification of Na^+^/K^+^ ATPase α1 subunit, which were heated to 65 °C according to the manufacturers recommendation) and loaded for 10 % acrylamide sodium dodecyl sulfate polyacrylamide gel electrophoresis (SDS-PAGE), submerged in electrophoresis buffer pH ~8 containing 25 mM Tris base 190 mM glycine 0.1 %. Proteins were then electrophoretically transferred to a polyvinyllidene difluoride membrane (Amersham) that had been incubated for 1 h in blocking solution containing of 5 % dried milk in Tris buffered saline (TBS; 150 mM NaCl 10 mM Tris pH 7.4) with 0.05 % Tween 20. Incubation in primary antibodies diluted in 5 % dried milk and Tris buffered saline: rabbit anti-Kir4.1, 1:1000 (Kalsi et al. 2004), rabbit anti-Kir5.1, 1:300 (Alomone), goat anti-Kir5.1, 1:300 (Santa Cruz), mouse anti-Na/K ATPase α1, 1:3000 (Abcam), mouse anti-PSD-95, 1:3000 (Thermo), whole serum anti-SAP-97, 1:500 (Amcam), rabbit anti-prohibitin, 1:300 (Abcam), goat anti-calnexin, 1:300 (Abcam). Following washes, incubation in horseradish peroxidase-conjugated secondary antibodies diluted in 5 % dried milk and Tris buffered saline was carried out for 1 h at RT: rabbit anti-goat, 1:3000 (Dako), goat anti-mouse, 1:10 000 (Dako), swine anti-rabbit 1:2000 (Dako). Extensive washing of the membranes in TBS with ice cold 0.05 % Tween20 was performed after each incubation and immunocomplexes were revealed using an enhanced chemiluminescence method (Amersham).

### Isolation of plasma membrane fraction

Whole brains and optic nerves of wild type and Kir4.1 KO animals were homogenised in subcellular fractionation buffer containing 250 mM sucrose, 20 mM HEPES, 10 mM KCl, 1.5 mM MgCl_2_, 1 mM EDTA, 1 mM EGTA, 1 DTT and 1× protease inhibitor cocktail. Samples were centrifuged at 4 °C with 750 G for 10 min to remove the nuclear fraction. Supernatant was placed in a new Eppendorf tube and centrifuged with 10,000*g* at 4 °C two times to remove the mitochondrial fraction. Supernatant was placed in an ultracentrifuge tube and centrifuged with 40,000*g* at 4 °C for 1 h. The supernatant was removed and the pellet containing the crude plasma membrane fraction was resuspended in 400 μl fractionation buffer using a 25 G needle and centrifuged at 40,000*g* at 4 °C for 45 min and then the pellet was resuspended in lysis buffer.

### Immunoprecipation

Brains and optic nerve obtained from adult wild type mice (P16–40) were homogenised as described above. For the precipitation of the proteins μMACS™ Protein A/G MicroBead kit was used (Miltenyi Biotec) according to the manufacturers recommendations. In brief, homogenised brain or optic nerve samples were centrifuged at 4 °C with 10,000*g* 2 times to clean the homogenate from cell debris. 3 µg polyclonal- or 2 µg monoclonal antibody was added to the proteins and incubated overnight at 4 °C. In the case of negative controls, proteins were mixed with the preabsorbed antibodies or for Kir4.1, Kir4.1 knock out tissue was used. Then, Protein G MicroBeads were added to the samples to magnetically label the immune complex (50 μl if monoclonal antibody was used or 100 μl if polyclonal antibody was used for the precipation). The samples were then mixed and incubated for 30 min on ice. The new μ Column were placed in the magnetic field of the μMACS™ separator and then prepared by rinsing with 200 μl of lysis buffer containing of 150 mM NaCl, 1 % Triton X-100, 50 mM Tris HCl (pH 8.0), before the cell homogenate was applied onto the column. The homogenate run through the columns was washed with 200 μl lysis buffer four times then with 100 μl of low-salt wash buffer containing of 1 % NP-40, 50 mM Tris HCl (pH 8.0). To prepare the elution of the immune complex 20 μl of pre-heated (95 °C) 1 × Laemelli sample buffer was applied onto the column and incubated for 5 min at RT. Next, 50 μl of pre-heated (95 °C) 1× Laemmli sample buffer was applied to the column matrix to elute the immune complex. The drop on the column tip containing the eluted immunoprecipitate was collected and stored on ice or at −80 °C until it was analyzed by SDS-PAGE.

## Results

### Oligodendrocytes and astrocytes express Kir4.1 and Kir5.1 in the brain

**Fig. 1:**
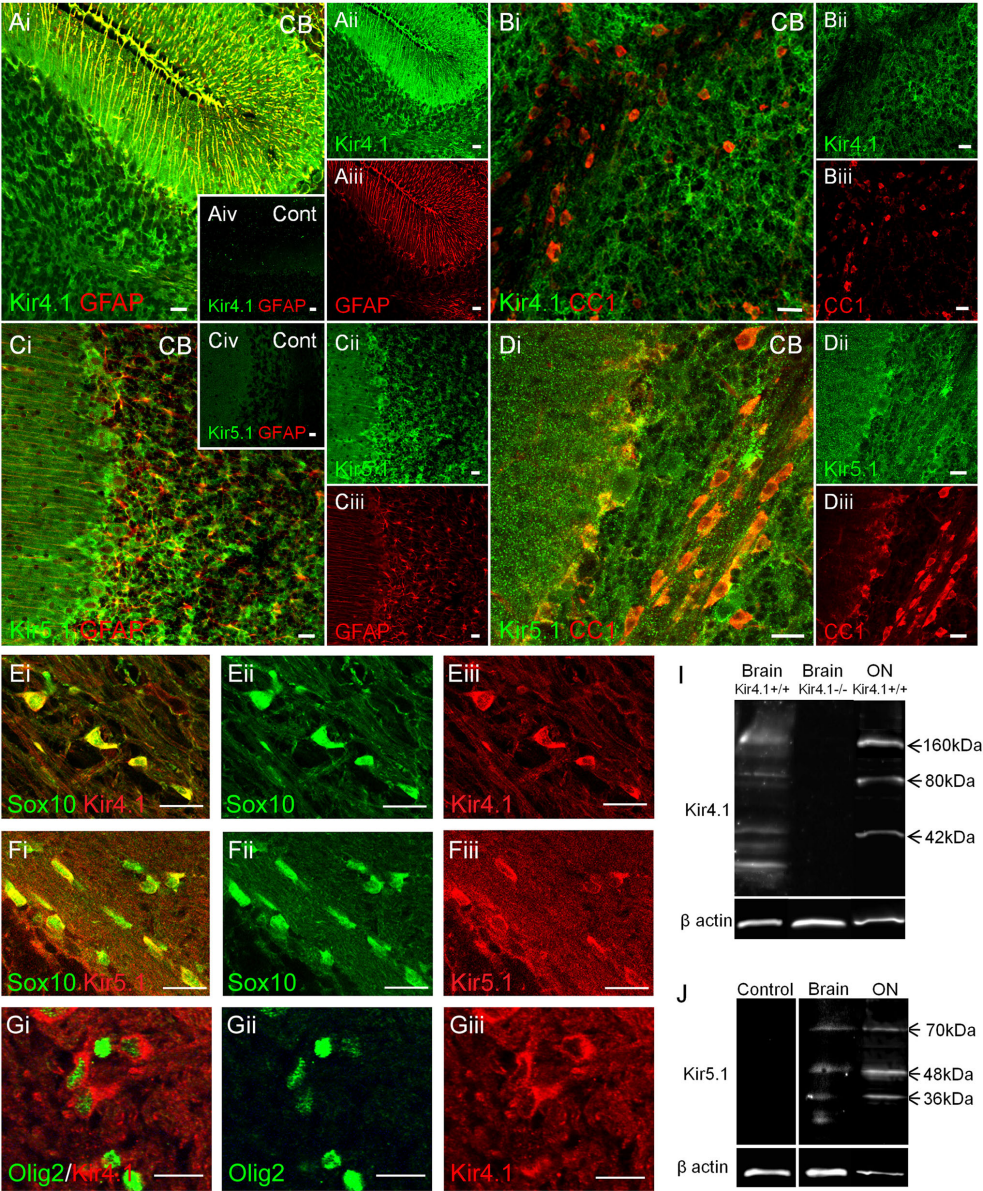
Expression of Kir4.1 and Kir5.1 in oligodendrocytes and astrocytes in the cerebellum. Immunolabelling for Kir4.1 and Kir5.1, in combination with GFAP for astrocytes (**A**, **C**), and APC/CC1 for oligodendrocytes (**B**, **D**). Immunolabelling for Kir4.1 (**E**) and Kir5.1 (**F**) in mice in which EGFP is under the control of the oligodendrocyte-specific Sox10 promoter. **G** Double immunolabelling for Kir4.1 (*red*) and the oligodenrocyte-specific marker Olig2 (*green*). *Insets* in *Aiv* and *Civ* illustrate negative controls, in the Kir4.1 KO mouse (*Aiv*) and following preincubation with the Kir5.1 blocking peptide (*Civ*). *Scale bars* 20 μm. Western blot analysis of the brain and optic and nerve for Kir4.1 (**I**) and Kir5.1 (**J**); bands were absent in the negative controls, in the Kir4.1 knock-out mouse (**I**) following preincubation in the Kir5.1 blocking peptide (**J**)

The Kir5.1 subunit forms functional channels as heteromers with Kir4.1, hence Kir5.1 expression might be expected to mirror that of Kir4.1 in the brain (Hibino et al. 2004). Kir4.1 is established as the main Kir subunit in astrocytes and is highly expressed in the cerebellum (Tang et al. 2009). In contrast, oligodendroglial expression of Kir4.1 is poorly defined, but they are reported to be immunopositive in the cerebellum (Poopalasundaram et al. 2000). Therefore, we assessed the expression of Kir5.1 in comparison with Kir4.1 in cerebellar astrocytes and oligodendrocytes (Fig. 1). Double immunofluorescence labeling demonstrated extensive colocalization between GFAP and Kir4.1, with strong and distinctive immunopositivity for Kir4.1 in the radial processes of Bergmann glia extending from the Purkinje cell layer through the molecular layer to the pia, together with astrocyte somata and processes in the granule cell layer and white matter (Fig. 1A). In contrast, the radial processes of Bergmann glia were not delineated by Kir5.1 immunolabelling, and Kir5.1 was most prominent in the granule cell layer and included distinct co-localisation with GFAP in astrocytes (Fig. 1C). The results indicate differential expression of Kir4.1 and Kir5.1 in Bergmann glia and cerebellar astrocytes. Expression of Kir4.1 and Kir5.1 in oligodendrocytes was examined using the APC(CC1) antibody (Bhat et al. 1996), Olig2 immunolabelling, and in sections from mice that express EGFP under the control of the oligodendrocyte-specific Sox10 promoter (Stolt et al. 2006); APC, Olig2 and Sox10 are essential for oligodendrocyte differentiation and their expression is routinely and extensively used to identify oligodendrocytes (Bhat et al. 1996; Bay and Butt 2013; Lang et al. 2013; Azim et al. 2014; Fancy et al. 2014). Although neurons also express APC, it does not appear to be recognized by the APC/CC1 antibody and may be a different isoform than found in oligodendrocytes (Brakeman et al. 1999). Double immunofluorescence labeling with APC(CC1) to label oligodendrocyte somata (Fig. 1B, D) and immunolabelling in Sox10-EGFP+ cells (Fig. 1E, F) demonstrates localization of Kir4.1 and Kir5.1 in oligodendrocytes of the cerebellar white matter. Further evidence of oligodendroglial expression of Kir4.1 is provided by immunolabelling with Olig2 (Fig. 1G). The results demonstrate robust expression of Kir4.1 and Kir5.1 in oligodendrocytes in the cerebellar white matter. No immunoreactivity was detected in negative controls in sections from knock out mouse for Kir4.1 (Fig. 1Aiv) and by preabsorbing the primary antibody with antigen peptide for Kir5.1 (Fig. 1Civ). In addition, the specificity of the antibodies was further confirmed by western blot analysis of total protein lysates in the brain and optic nerve (Fig. 1I, J). For Kir4.1, the predicted molecular weights of the monomeric 42 kDa, heteromeric 80 kDa, and tetrameric 160 kDa channels were identified in wild-type mice, and these were absent in brain lysates from Kir4.1 knock-out mice (Fig. 1I). Western blot for Kir5.1 was performed in wild type mice, with a predicted molecular weight of 48 kDa, together with bands at 36 kDa and 70 kDa, depending on the extent of glycosylation of the cytoplasmic and membrane bound channels (Hibino et al. 2004), and positive bands were eliminated by the competitive peptide (Fig. 1J).

### Localization of Kir4.1 and Kir5.1 in optic nerve astrocytes and oligodendrocytes

**Fig. 2:**
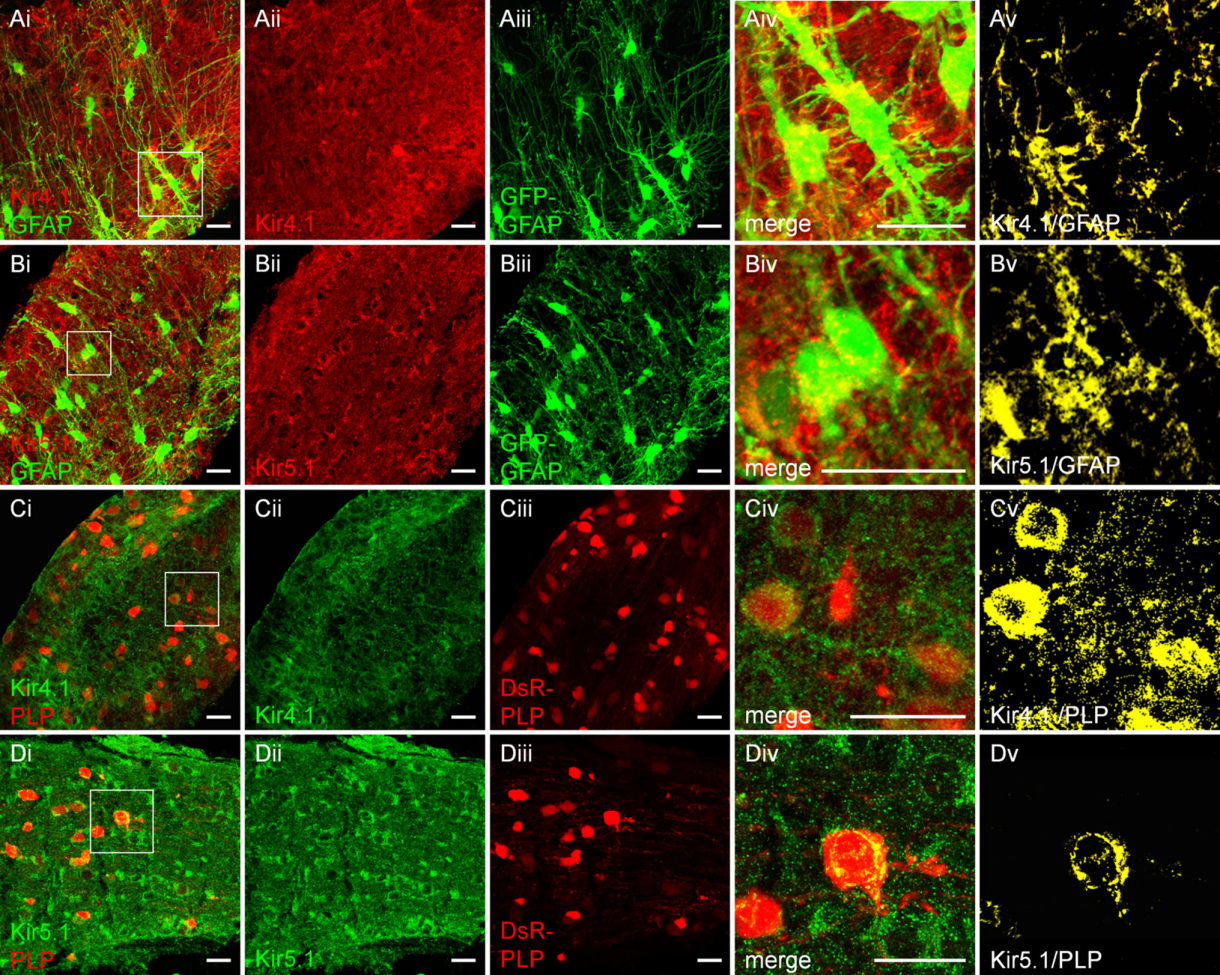
Expression of Kir4.1 and Kir5.1 in optic nerve oligodendrocytes and astrocytes. Immunolabelling for Kir4.1 (**A**, **C**) and Kir5.1 (**B**, **D**), in GFAP-GFP mice to identify astrocytes (**A**, **B**) and PLP-DsRED mice to identify oligodendrocytes (**C**, **D**). Cellular expression of Kir4.1 and Kir5.1 is demonstrated by the generation of colocalisation channels (**Av**, **Bv**, **Cv**, **Dv**) from confocal *z*-stacks (**Aiv**, **Biv**, **Civ**, **Div**), and *green* and *red* channels of equal intensity appear *yellow*. *Scale bars* 20 μm

The optic nerve is a typical myelinated CNS white tract and immunolocalization of Kir4.1 has been clearly identified in astrocytes and oligodendrocytes (Kalsi et al. 2004). We therefore examined the relationships between Kir5.1 and Kir4.1 in optic nerve glia, using transgenic reporter mice in which EGFP is under the control of the astrocyte-specific promoter for the human GFAP gene and DsRed is under the control of the oligodendrocyte-specific PLP gene (Fig. 2). Extensive immunostaining for Kir4.1 and Kir5.1 was localised both in transversely oriented astrocytes (Fig. 2A, B) and longitudinal rows of oligodendrocytes (Fig. 2C, D). Colocalization of fluorescence labelling is most frequently presented as overlays of red and green channels, with areas of yellow indicating colocalization (Fig. 2Aiv, Biv, Civ, Div). To analyse this more accurately, we used the technique of Barlow and colleagues (Barlow et al. 2010), to perform quantification of the degree of colocalization between Kir4.1 and Kir5.1 with GFAP-EGFP and PLP-DsRed on high resolution confocal *z*-sections (Bay and Butt 2013). In brief, the background intensity measured in negative control sections (averaged from a minimum of 6 images) was used to threshold the images and determine the Pearson’s correlation coefficients in single *z*-sections, from which colocalization channels were generated. In this way, the colocalization channel identifies the individual voxels in which the two channels overlap with the same intensity (Fig. 2Av, Bv, Cv, Dv). The results demonstrate optic nerve astrocytes and oligodendrocytes express Kir5.1 and Kir4.1 throughout their cell somata and processes.

### Plasmalemmal expression of Kir4.1 and Kir5.1 subunits in optic nerve glia

**Fig. 3:**
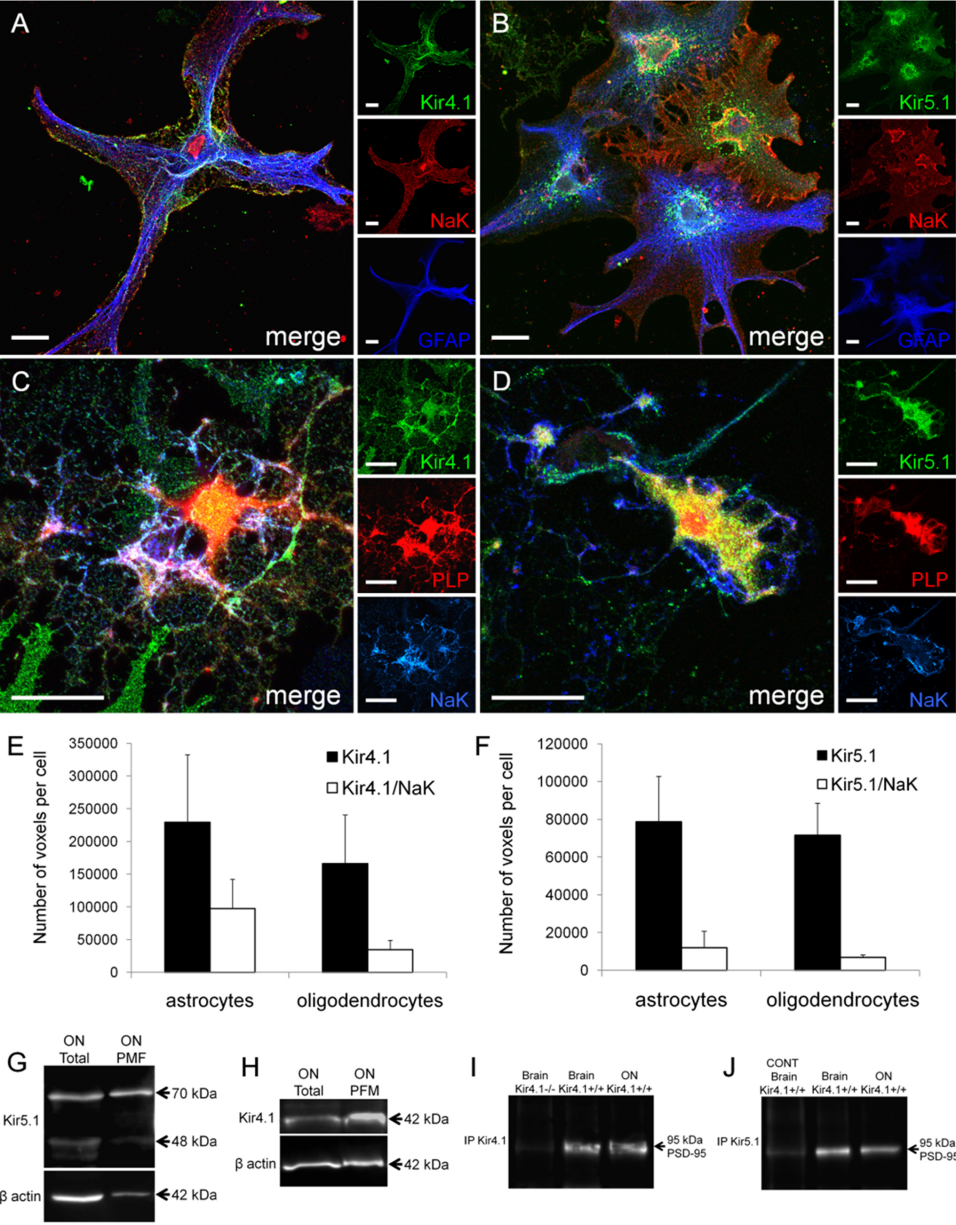
Plasmalemmal expression of Kir4.1 and Kir5.1 subunit in optic nerve glia. Immunolocalization of Kir4.1 and Kir5.1 with the membrane bound Na–K-ATPase α1 subunit in optic nerve explants of astrocytes identified by GFAP (**A**, **B**) and oligodendrocytes identified by PLP-DsRed (**C**, **D**). *Scale bars* 20 μm. Quantification in astrocytes and oligodendrocytes of total number of voxels immunopositive for Kir4.1 and Kir5.1, compared to voxels that were identified as colocalized for Kir4.1/Na–K-ATPase (**E**) and Kir5.1/Na–K-ATPase (**F**); data are mean ± SEM, *n* = 13 cells for each analysis. Western blot analysis of Kir5.1 (**G**) and Kir4.1 (**H**) in total optic nerve lysate and plasma membrane fraction. Co-immunoprecipitation of Kir4.1 (**I**) and Kir5.1 (**J**) with PSD95, in total brain and optic nerve (ON) lysate; negative controls were Kir4.1 knock-out mice (−/−) for Kir4.1 and preincubation with the blocking peptide for Kir5.1

To be functional, Kir4.1 and Kir5.1 subunits need to be expressed in the plasmalemma and this was assessed by two methods: immunocolocalization with the plasma membrane marker Na^+^–K^+^-ATPase α1 subunit in optic nerve glial explant cultures, and western blot analysis of purified plasma membrane fractions (Fig. 3). Immunolabelling of optic nerve explant cultures demonstrates Kir4.1 and Kir5.1 colocalise with Na^+^–K^+^-ATPase in both GFAP-positive astrocytes (Fig. 3A, B) and PLP-positive oligodendrocytes (Fig. 3C, D). Quantification of co-localization, as described above, demonstrated significant co-localisation of both Kir4.1 and Kir5.1 with Na^+^–K^+^-ATPase in astrocytes and oligodendrocytes (Fig. 3E, F). Western blot analysis of plasmamembrane fractions compared to total lysates confirmed plasmalemmal expression of Kir5.1 and Kir4.1 subunits in the optic nerve (Fig. 3G, H). Measurement of the density of the bands relative to β-actin indicated a 3.9-fold enrichment of Kir5.1 in the optic nerve plasma membrane fraction, compared to 2.1-fold enrichment for Kir4.1; no bands were observed in the negative controls, using the Kir4.1 knock-out mouse and pre-incubation in the Kir5.1 blocking peptide. In addition, PSD-95 clusters Kir channels in the cell membrane through its interaction with their PDZ domains (Horio et al. 1997; Tanemoto et al. 2002; Pegan et al. 2007), hence we used co-immunprecipitation of total brain and optic nerve lysates to determine whether PSD-has a role in anchoring Kir4.1 and Kir5.1 in optic nerve glia (Fig. 3I, J). PSD-95 was co-immunoprecipitated with both Kir4.1 (Fig. 3I) and Kir5.1 (Fig. 3J) in the brain and optic nerve; western blot of the anti-PSD-95 antibody gave a dense band at the predicted MW and no band was detected in the negative controls, and no co-immunoprecipitiation with PSD-95 was observed in the negative controls, using the knock-out mouse for Kir4.1 (Fig. 3I) and blocking peptide for Kir5.1 (Fig. 3J). These results demonstrate Kir4.1 and Kir5.1 subunit expression in the glial plasmalemma and support a role for PSD-95 in their membrane localization.

### Co-localization of Kir4.1 and Kir5.1 in astrocytes and oligodendrocytes

**Fig. 4:**
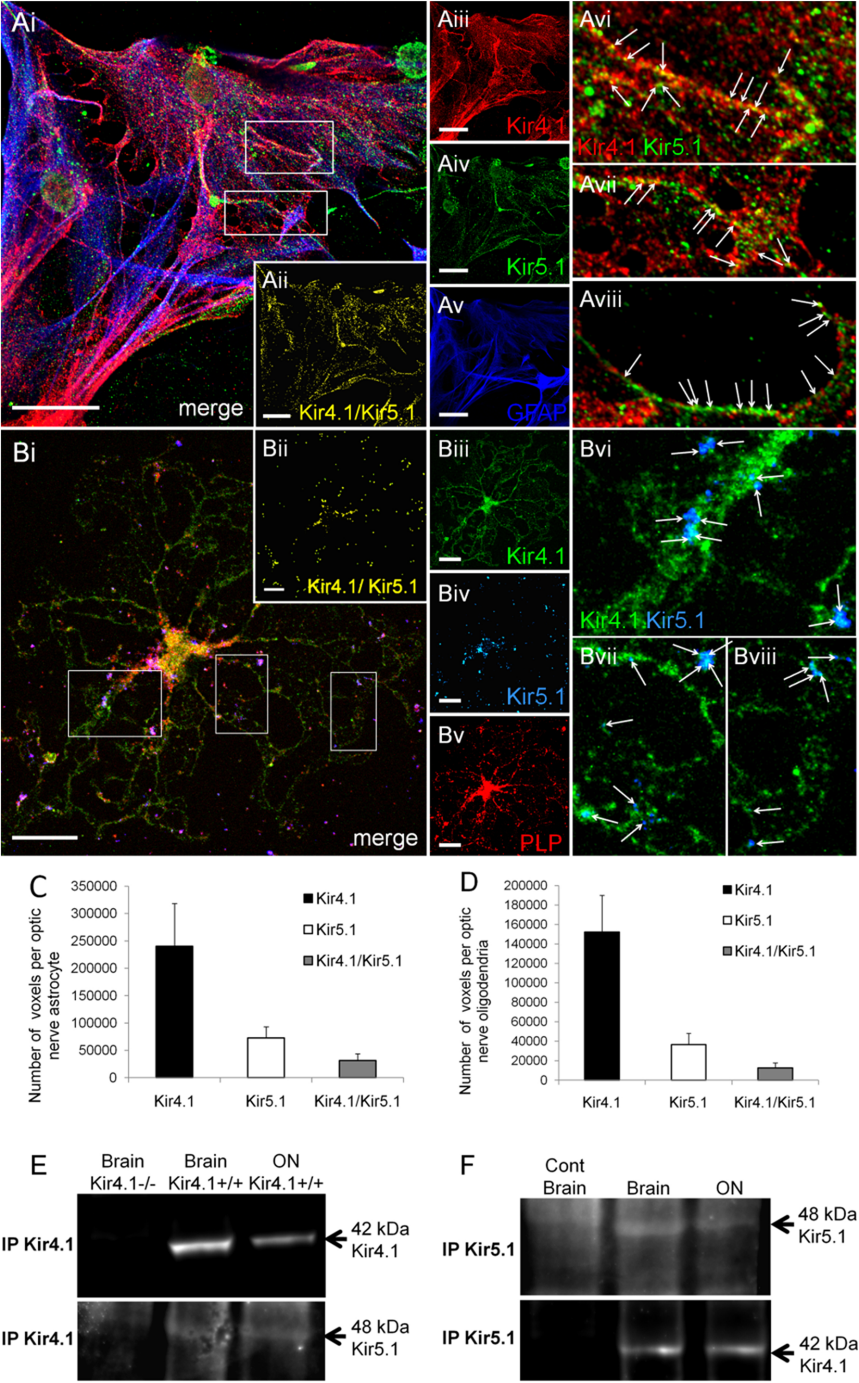
Co-expression of Kir4.1 and Kir5.1 in optic nerve oligodendrocytes and astrocytes. Co-immunolocalization of Kir4.1 and Kir5.1 in optic nerve explant cultures, in astrocytes identified by GFAP immunolabelling (**A**) and oligodendrocytes identified by PLP-DsRED (**B**). The overlay and individual channels are illustrated, together with the co-localisation channel for Kir4.1/Kir5.1 (**Aii, Bii**). *Boxed areas* on overlay images (**Ai**, **Bi**) are enlarged in *Avi*–*Aviii* and *Bvi*–*Bviii*, to illustrate punctate colocalization of Kir4.1 and Kir5.1 along processes (some indicated by *arrows*). *Scale bars* 20 μm. Quantification of the number of voxels that were positive for Kir4.1 and Kir5.1 alone and of Kir4.1/Kir5.1 together, in astrocytes (**C**, *n* = 15) and oligodendrocytes (**D**, *n* = 13); data are mean ± SEM. Co-immunoprecipitation of Kir4.1 with Kir5.1 (**E**) and of Kir5.1 with Kir4.1 (**F**) from total brain and optic nerve (ON) lysates; negative controls were Kir4.1 knock-out mice (−/−) for Kir4.1, and using the blocking peptide for Kir5.1

Unequivocal cellular colocalization of Kir4.1 and Kir5.1 was demonstrated in vitro, by double immunofluorescence labelling with rabbit anti-Kir4.1 and goat anti-Kir5.1 antibodies in GFAP-positive astrocytes (Fig. 4Ai) and PLP-positive oligodendrocytes (Fig. 4Bi). Co-localisation analysis indicated both Kir4.1 homomeric and Kir4.1/Kir5.1 heteromeric channels in astrocytes (Fig. 4C) and oligodendrocytes (Fig. 4D), and this was confirmed by co-immunoprecipitation in brain and optic nerve extracts, in both directions, with Kir5.1 protein being detected in the Kir4.1 immunoprecipitate (Fig. 4E, lower lane), and Kir4.1 being detected in the Kir5.1 immunoprecipate (Fig. 4F, lower lane); immunoblotting was absent in negative controls, using the knock out mouse for Kir4.1 (Fig. 4E) and preincubation with the blocking peptide for Kir5.1 (Fig. 4F). The results were confirmed in 5 different experiments and demonstrate that optic nerve glia express heteromeric Kir4.1/Kir5.1 channels. In addition, there was punctate Kir5.1 immunostaining that was not co-localised with Kir4.1 within the cytoplasm of the somata and along the processes of astrocytes (Fig. 4Avi–viii, arrows) and oligodendrocytes (Fig. 4Bvi–viii, arrows).

### Glial Kir5.1 is downregulated in the absence of Kir4.1

**Fig. 5:**
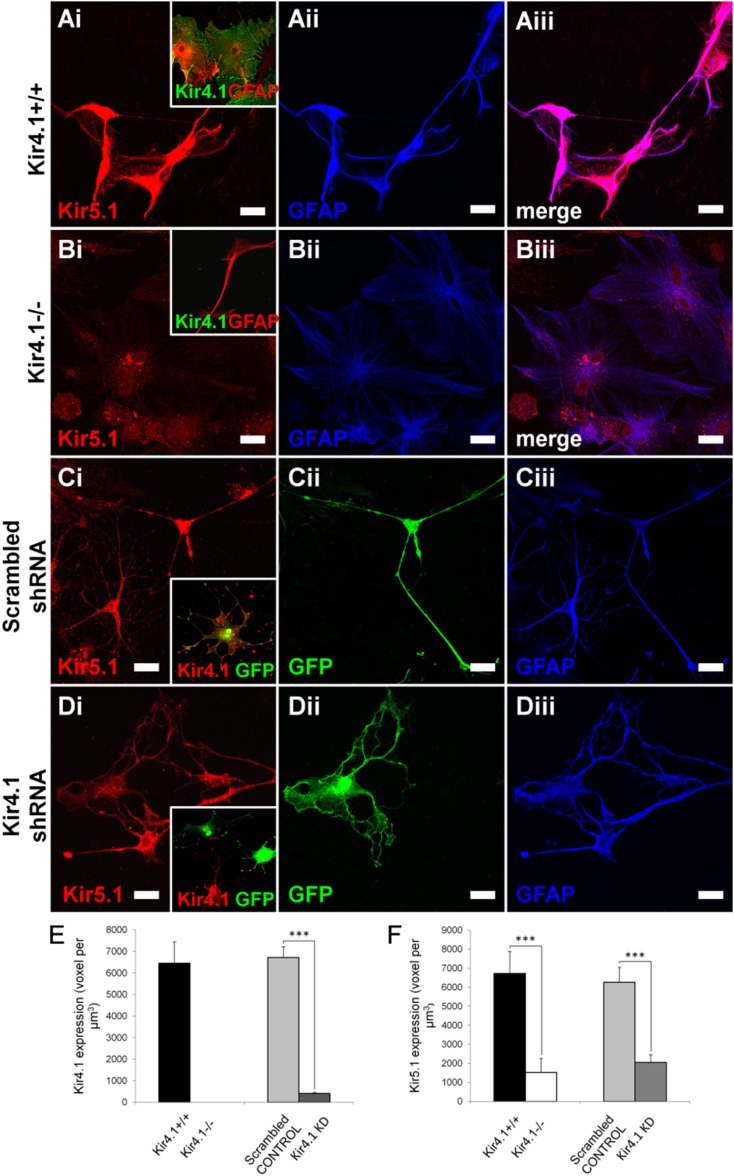
Glial Kir5.1 expression is reduced in the absence of Kir4.1 subunit. Immunolabelling for Kir5.1 was determined in optic nerve explants cultures, comparing wild-type mice (**A**, Kir4.1^+/+^) with Kir4.1 knock-out mice (**B**, Kir4.1^−/−^), and following transfection with scrambled shRNA (**C**) or Kir4.1 shRNA (**D**); transfected cells were identified by the expression of GFP (appears *green*) and *insets* demonstrate Kir4.1 expression in controls (**Ai**, **Ci**) and complete ablation in Kir4.1^−/−^ mice (**Bi**) and Kir4.1 shRNA (**Di**). *Scale bars* 10 μm. Quantification of expression of Kir4.1 (**E**) and Kir5.1 (**F**) in Kir4.1^+/+^, Kir4.1^−/−^, scrambled control and Kir4.1shRNA glia; analysis was performed on 10–12 cells in each group, and data are expressed as mean ± SEM number of voxels per µm^3^, ***p < 0.001, one-tailed *t* test

The results presented above demonstrate astrocytes and oligodendrocytes express heteromeric Kir4.1/Kir5.1 channels. To evaluate this further, we compared Kir5.1 immunolabelling in optic nerve explants from Kir4.1 +/+ wild type mice (Fig. 5A) and Kir4.1^−/−^ knock out mice (Fig. 5B), and using shRNA to knock down Kir4.1 in optic nerve glial explant cultures (Fig. 5D), compared to scrambled shRNA vector in controls (Fig. 5C). We established above that Kir4.1 is absent in brains and optic nerves of Kir4.1 knock-out mice (Fig. 1). We found equivalent results in optic nerve explants, with robust Kir4.1 immunolabelling in glia from wild-type mice (Fig. 5Ai, inset) and absence of Kir4.1 immunolabelling in cells from Kir4.1 knock-out mice (Fig. 5Bi, inset), which was confirmed by quantification (Fig. 5E). Similarly, Kir5.1 immunolabelling was strong in wild-type cells (Fig. 5A), but in the absence of Kir4.1, there was a marked decrease in Kir5.1 immunolabelling (Fig. 5B), which was statistically significant (Fig. 5F). In addition, we demonstrate that knock-down of Kir4.1 with shRNA significantly reduced the expression of Kir5.1 (Fig. 5D), whereas controls were unaffected (Fig. 5C), as summarised in the histograms (Fig. 5F); transfected cells were identified by GFP expression and Kir5.1 expression was not significantly different between wild-type and scrambled controls, or between Kir4.1 knock out and Kir4.1 shRNA cells (Fig. 5F). We confirmed the loss of Kir4.1 immunolabelling following Kir4.1 knock-down (Fig. 5Di, inset), whereas transfection with scrambled shRNA had no effect on Kir4.1 (Fig. 5Ci, inset), and the results were statistically significant (Fig. 5E).

### Plasmalemmal Kir5.1 is decreased in the absence of Kir4.1

**Fig. 6:**
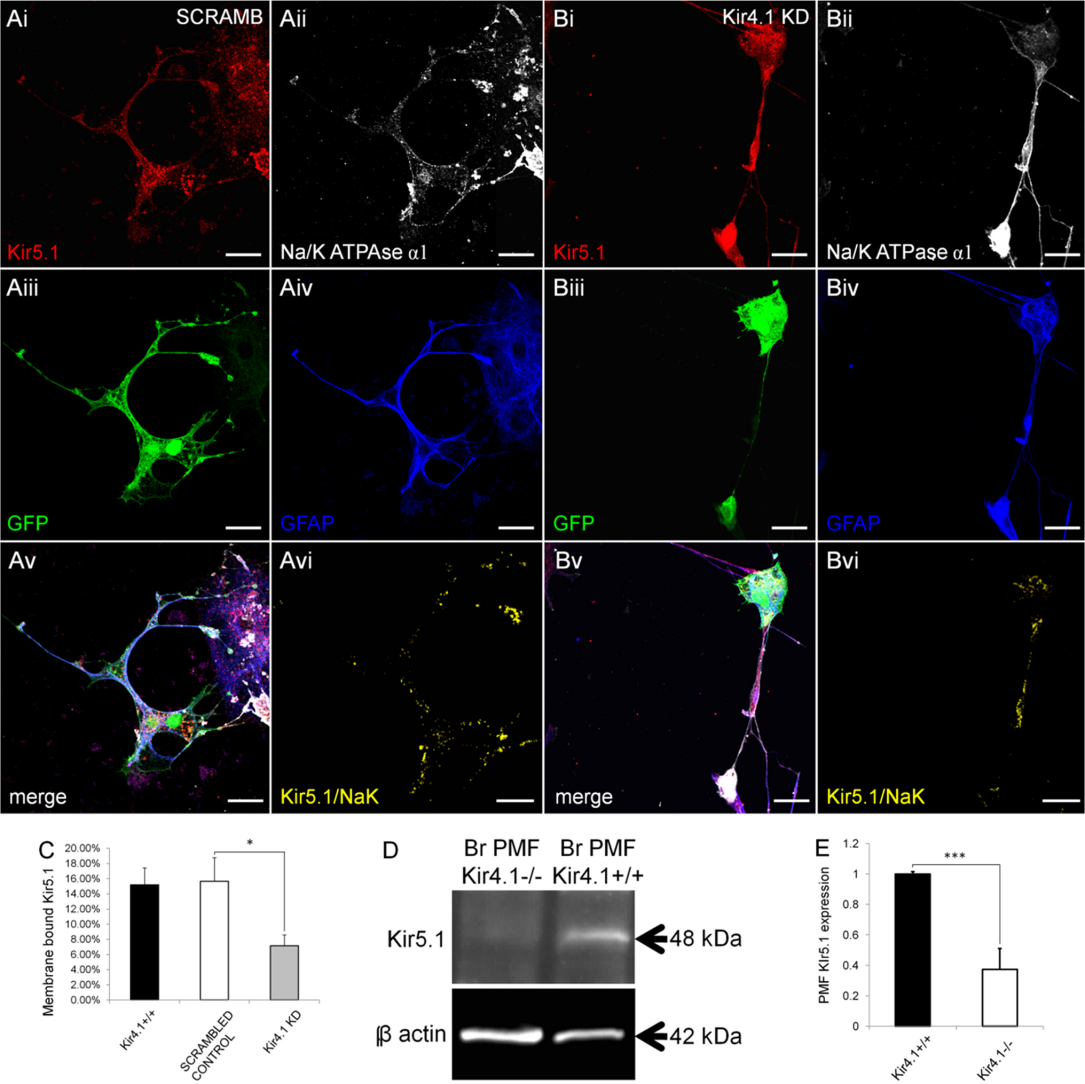
Specific reduction in plasmalemmal Kir5.1 in the absence of Kir4.1. Immunolocalization of Kir5.1 with the membrane bound Na–K-ATPase α1 subunit in optic nerve explant astrocytes identified by expression of GFAP, following transfection with scrambled shRNA (**A**) or Kir4.1 shRNA (**B**); transfected cells were identified by co-transfection with GFP (appears *green*) and the co-localization channel indicates voxels in which Kir5.1 and Na–K-ATPase immunolabelling was at the same intensity (**Avi**, **Bvi**). *Scale bars* 20 μm. **C** Quantification of plasmalemmal Kir5.1 expressed as percentage of total Kir5.1 + voxels (data are mean ± SEM, *n* = 11–13 per group; *p < 0.05, one-tailed *t* test). **D**, **E** Western blot of Kir5.1 expression in the brain plasma membrane fraction from Kir4.1^+/+^ wild-type and Kir4.1^−/−^ knock-out mice (**D**) and mean (±SEM) integrated density normalised against β-actin (**E**, *n* = 3, ***p < 0.001, one-tailed *t* test)

Immunocolocalization with the plasma membrane marker Na^+^–K^+^-ATPase α1 subunit (Fig. 6A–C), and western blot analysis of the plasmamembrane fraction (Fig. 6D) demonstrates a loss of plasmalemmal Kir5.1 in the absence of Kir4.1. As in non-transfected cells shown above (Fig. 3A–D), there was extensive co-localization of Kir5.1 and Na^+^–K^+^-ATPase in controls transfected with scrambled shRNA (Fig. 6A), whereas co-localisation was significantly reduced following transfection with Kir4.1 shRNA (Fig. 6B), resulting in a twofold decrease in Kir5.1/Na^+^–K^+^-ATPase colocalized voxels from 15.2 % in controls to 7.1 % following Kir4.1 knock-down (Fig. 6C). Similarly, western blot of the brain plasma membrane fraction from wild-type and Kir4.1 knock-out mice demonstrated a significant reduction in Kir5.1 levels by 60 ± 14 % in the absence of Kir4.1 (Fig. 6D, E). These results demonstrate an overall decrease in Kir5.1 expression in the absence of Kir4.1 and a specific reduction in plasmalemmal Kir5.1, providing evidence that the bulk of glial Kir5.1 is expressed in the form of heteromeric channels with Kir4.1, and most of the remaining Kir5.1 subunits appear to be retained intracellulary and do not form plasmalemmal channels in the absence of the Kir4.1.

### Kir5.1 is decreased in myelin in the absence of Kir4.1

**Fig. 7:**
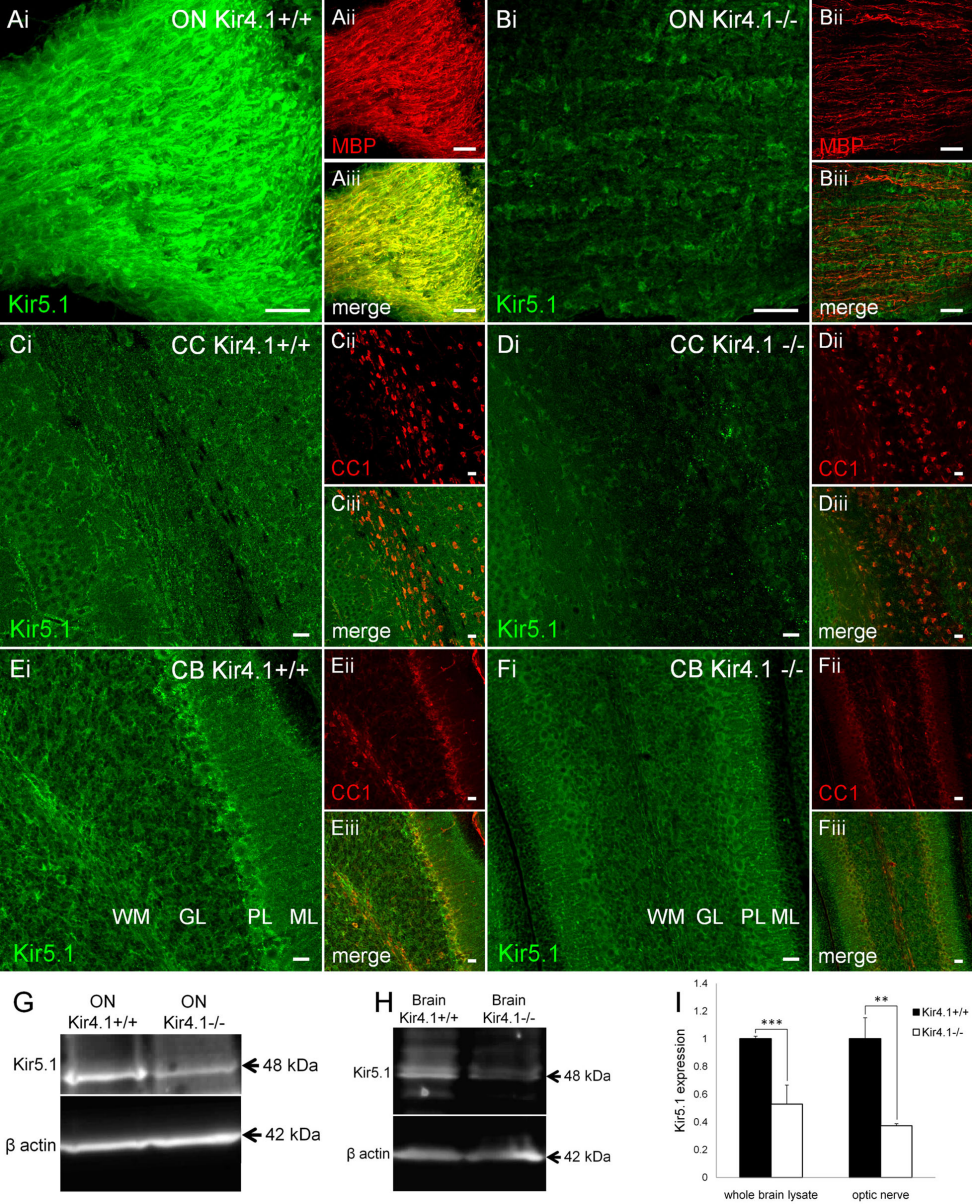
Reduction of Kir5.1 in oligodendrocytes and myelin in the absence of Kir4.1. Immunolocalization of Kir5.1 with myelin basic protein, MBP (**A**, **B**) and the oligodenrocyte marker APC/CC1 (**C**–**F**), in brain tissue from wild-type Kir4.1^+/+^ mice (**A**, **C**, **E**) compared to Kir4.1^−/−^ knock-out mice (**B**, **D**, **F**). *Scale bars* 20 μm. Western blot analysis of Kir5.1 from total lysates of optic nerve (**G**) and brain (**H**) from wild-type Kir4.1^+/+^ and Kir4.1^−/−^ knock-out mice, and mean (±SEM) integrated density normalised against β-actin (**I**, *n* = 3, **p < 0.01; ***p < 0.001, one-tailed *t* test)

Oligodendrocytes were very rarely transfected in our in vitro experiments and so we examined interactions between Kir5.1 and Kir4.1 in oligodendrocytes by immunolabelling of optic nerve and brain sections from Kir4.1 knock-out mouse. There is a complete loss of Kir4.1 immunolabeling in the knock-out mouse (Fig. 1Aiv), and this results in an evident decrease in Kir5.1 immunolabelling overall, as well as a specific decrease in Kir5.1 in oligodendrocytes and myelin, as illustrated by MBP immunolabelling in the optic nerve (Fig. 7A, B), and by APC(CC1) immunolabeling in the corpus callosum (Fig. 7C, D) and cerebellum (Fig. 7E, F). We showed above by western blot a complete loss of Kir4.1 protein in the knock-out mouse (Fig. 1I), and this resulted in significant decreases in Kir5.1 protein levels in the optic nerve (Fig. 7G) and brain (Fig. 7H), by 33.5 ± 3.5 and 53 ± 0.4 %, respectively (Fig. 7I; *p* < 0.001, unrelated *t* test).

## Discussion

The inward rectifying K^+^ channel subtype Kir4.1 has been considered to be almost exclusively astroglial in the CNS (Hibino et al. 2004; Tang et al. 2009), and forms heteromeric channels with the Kir5.1 subunit in astrocytes from a number of brain regions (Ishii et al. 2003; Hibino et al. 2004). Here, we demonstrate that oligodendrocytes, the myelinating cells of the CNS, express plasmalemmal homomeric Kir4.1 channels and heteromeric Kir4.1/Kir5.1 channels. Oligodendroglial expression of Kir4.1 has been considered controversial (Tang et al. 2009), and is likely to be heterogeneous (Poopalasundaram et al. 2000). Our study demonstrates that Kir4.1 immunolabelling is colocalised with multiple markers for oligodendrocytes: (1) the PLP-DsRed reporter, (2) Sox10-eGFP reporter, (3) APC(CC1) and (4) Olig2. Kir4.1 and Kir4.1/Kir5.1 channels have distinct functions in astroglial K^+^ transport and pH/CO_2_ chemosensation (Mulkey and Wenker 2011; Kofuji and Newman 2004; Butt and Kalsi 2006; Olsen and Sontheimer 2008). Our results indicate the distinct properties of these channels are important for the oligodendrocyte function of myelination.

### Oligodendrocytes and white matter astrocytes express Kir4.1 and Kir4.1/5.1

Co-immunoprecipitation and immunolabeling identified the existence of both homomeric Kir4.1 and heteromeric Kir4.1/Kir5.1 channels in astrocytes and oligodendrocytes of optic nerve and cerebellar white matter. The anti-Kir4.1 antibodies used in this study have been validated previously (Kalsi et al. 2004) and their specificity was confirmed using western blot and immunohistochemistry in Kir4.1 knock-out mice, in support of previous studies using a similar antibody (Kofuji et al. 2000; Neusch et al. 2001). In the case of Kir5.1, the specificity of the antibodies was confirmed by using blocking peptides and comparing two commercially available antibodies from Alomone (rabbit anti-rat Kir5.1 antibody) and Santa Cruz (goat anti-mouse Kir5.1 antibody), raised againist two different non overlapping epitopes in the C-terminus of the channel. The two antibodies provided equivalent immunoblot and immunolabeling results that were completely absent in the presence of blocking peptide. Expression of Kir4.1 and Kir5.1 in oligodendrocytes was verified in three ways: (1) in double immunolabelling with the anti-APC(CC1) and Olig2 antibodies—APC and Olig2 are essential for oligodenrocyte differentiation and the CC1 antibody is used routinely and extensively to identify oligodendrocytes in the CNS (Bhat et al. 1996; Bay and Butt 2013; Lang et al. 2013; Azim et al. 2014; Fancy et al. 2014); (2) immunolabelling in sections from mice that express EGFP under the control of the Sox10 promoter, which is a transcription factor that is essential for oligodendrocyte differentiation and is expressed by all oligodendrocyte lineage cells (Stolt et al. 2006; Azim et al. 2014); (3) immunolabelling in sections and isolated cells from mice that express DsRed under the control of the myelin protein PLP (Hirrlinger et al. 2005; Azim et al. 2014). The results demonstrate expression of Kir4.1 and Kir5.1 in astrocytes and oligodendrocytes, and specifically plasmalemmal localisation of the Kir subunits on glia by immunoblot of optic nerve plasma membrane and immunocytochemical co-localisation with plasma membrane-bound Na–K-ATPase. Immunoblot analysis demonstrated robust bands as predicted according to the momomer subunit molecular weights, 42 kDa for Kir4.1 and 48 kDa for Kir5.1. In addition, bands for Kir4.1 were indicated at 80 and 160 kDa, which corresponds to the heteromeric and homo-tetrameric channel as observed previously in the brain and retina (Kofuji et al. 2000; Ulbricht et al. 2008). Furthermore, bands were detected at 70 kDa for Kir5.1, which were more enriched in the optic nerve plasma membrane fraction, and are consistent with previous findings indicating the ion channel is regulated by glycosylation (Ishii et al. 2003). A key finding was that the co-immunoprecipitation and immunocytochemical analyses indicated oligodendrocytes and astrocytes express Kir4.1/Kir5.1 heteromers, with the majority of Kir5.1 being associated with Kir4.1 and present in the plasmalemma, which is essential for functional heteromeric channels. Furthermore, examination of Kir4.1 knock-out mouse tissue and using shRNA to ablate Kir4.1 in vitro demonstrated that plasmalemmal Kir5.1 expression was almost completely dependent on Kir4.1. In contrast, the bulk of Kir4.1 (>80 %) in optic nerve glia was not co-expressed with Kir5.1, indicating a predominance of homomeric Kir4.1 channels.

### Heterogeneity of glial Kir4.1 and Kir5.1 expression

Astroglial Kir4.1 and Kir5.1 immunolabelling was heterogeneous in the brain and optic nerve, indicating that most astrocytes expressed Kir4.1 and Kir5.1 to a high degree, but a subpopulation were only weakly immunopositive or possible immunonegative for Kir5.1, in agreement with previous studies (Poopalasundaram et al. 2000; Hibino et al. 2004; Kalsi et al. 2004; Tang et al. 2009). In the cerebellum, Bergmann astroglia were distinctive in that their primary processes were strongly decorated with Kir4.1, but not Kir5.1, whereas astrocytes in the granule cell layer expressed Kir4.1 and Kir5.1. White matter astrocytes were enriched with Kir4.1, which is consistent with studies in knock-out mice demonstrating Kir4.1 are essential for prominent astroglial inward currents and the uptake of K^+^ released by axons during action potential propagation (Kofuji et al. 2000; Neusch et al. 2006; Djukic et al. 2007; Seifert et al. 2009; Bay and Butt 2013). A prominent role for heteromeric Kir4.1/Kir5.1 channels in K^+^ transport has also been demonstrated in transporting epithelia, for example in the renal tubules and gastric parietal cells (Paulais et al. 2011; Tanemoto et al. 2000; Tucker et al. 2000; Lachheb et al. 2008). Heteromeric Kir4.1/Kir5.1 channels are stronger inward rectifiers than homomeric Kir4.1 channels, which provides a potential mechanism for the uptake of K^+^ at sites of high activity via Kir4.1/Kir5.1 and release of K^+^ at sites of low activity via homomeric Kir4.1 channels (Kofuji et al. 2002; Butt and Kalsi 2006; Bay and Butt 2013). The close association of Kir4.1 and Kir5.1 with plasmalemmal Na^+^–K^+^-ATPase suggests the targeting of these channels to the same membrane microdomains may help provide K^+^ for the activity of the pumps, as observed for H^+^–K^+^-pumps in parietal cells (Fujita et al. 2002; Kaufhold et al. 2008). In the CNS, heterometric Kir4.1/Kir5.1 channels have been described in retinal Müller cells, brain astrocytes derived from neocortex, retrotrapezoid nucleus and the glomeruli of the olfactory bulb, and brainstem neurons from the cardio-respiratory nuclei (Hibino et al. 2004; Ishii et al. 2003; Tanemoto et al. 2000; Tucker et al. 2000; Wu et al. 2004; Yamamoto et al. 2008). Interestingly, a study using a bacterial artificial chromosome (BAC) transgenic approach to express EGFP under the transcriptional control of the Kir4.1 promoter reported prominent expression of EGFP in astrocytes but not oligodendrocytes (Tang et al. 2009). In contrast, Kir4.1 have been detected in oligodendrocytes by immunohistochemistry (Poopalasundaram et al. 2000; Kalsi et al. 2004) and electrophysiological experiments demonstrate prominent Kir in oligodendrocytes (Neusch et al. 2001; Bolton and Butt 2006), and genetic ablation of Kir4.1 results in a loss of Kir currents and causes severe dysmyelination (Neusch et al. 2001). Our results demonstrate that in white matter of the optic nerve and cerebellum oligodendrocytes express heteromeric Kir4.1/Kir5.1 channels, as well as homomeric Kir4.1 channels, and we show the loss of Kir4.1 is associated with a marked reduction of Kir5.1 in oligodendrocytes and myelin, supporting a role for these channels in oligodendroglial K^+^ regulation and myelin integrity (Neusch et al. 2001; Bolton and Butt 2006; Menichella et al. 2006). The discrepancy in the literature is likely to reflect the heterogeneity of oligodendroglial expression of Kir4.1, which appears greatest in the optic nerve, cerebellum and spinal cord, whereas in the forebrain Kir4.1 is far more prominent in protoplasmic astrocytes compared to oligodendrocytes (Poopalasundaram et al. 2000; Neusch et al. 2001; Kalsi et al. 2004).

### Dependence of glial Kir5.1 expression on the Kir4.1 subunit

Genetic ablation of Kir4.1 or knock-down using shRNA demonstrated that plasmalemmal expression of Kir5.1 in astrocytes and oligodendrocytes was largely dependent on its association with Kir4.1, consistent with clustering of Kir5.1 in the plasma membrane through the PDZ domain of the Kir4.1 subunit, which prevents internalisation of Kir5.1 (Konstas et al. 2003; Tanemoto et al. 2004). In addition, we provide evidence that clustering of Kir4.1 and Kir5.1 involves the MAGUK family PDZ-binding anchoring proteins PSD-95, which has been described in other cells (Horio et al. 1997; Tanemoto et al. 2002). Although Kir5.1 homomeric channels are generally thought to be non-functional, there is evidence that clustering of Kir5.1 subunits by PSD-95 mediates the formation of functional homomeric Kir5.1 channels in the brain (Tanemoto et al. 2002). We observed co-immunoprecipitation of Kir5.1 with PSD-95 in the optic nerve and a high degree of Kir5.1 immunolabelling in optic nerve glia was not associated with Kir4.1, raising the possibility they may express functional homomeric Kir5.1 channels. However, Kir5.1 may also form heteromers with other Kir subtypes, including Kir2.1 (Derst et al. 2001; Pessia et al. 2001), which is expressed by astrocytes (Horio 2001; Howe et al. 2008; Kang et al. 2008), and also clusters with PSD-95 (Fomina et al. 2011; Nehring et al. 2000; Leonoudakis et al. 2001; Pegan et al. 2007). Our results identified that Kir5.1 immunolabeling was mostly concentrated within the cell cytoplasm, tenfold greater than at the plasmalemma, indicating they provide a pool by which plasmallemal Kir4.1/Kir5.1 channels could be inserted into the cell membrane and provide dynamic regulation of the glial membrane potential in response to changes in the extracellular environment (Bolton and Butt 2006; Bolton et al. 2006). Kir5.1 are known to display intracellular localization and phosphorylation dependent interactions result in the targeting of Kir5.1/Kir4.1 heteromeric channels to the membrane (Tanemoto et al. 2000, 2004, 2008). Furthermore, Kir4.1/Kir5.1 channels are regulated by multiple factors, including PKA, PIP2, pH and CO_2_ (Xu et al. 2000; Yang et al. 2000; Rapedius et al. 2007; Rojas et al. 2007, 2008). Hence, the cytoplasmic pool of Kir5.1 would allow astrocytes and oligodendrocytes to respond rapidly to changes in the extracellular environment and dynamically target heteromeric Kir4.1/Kir5.1 channels to the cell membrane to provide channels with much stronger rectification and pH/CO_2_ sensitivity than Kir4.1 homomeric channels (Tanemoto et al. 2000; Yang et al. 2000; Pessia et al. 2001).

### Functional implications of heteromeric Kir4.1/Kir5.1 channels in oligodendrocytes

**Fig. 8:**
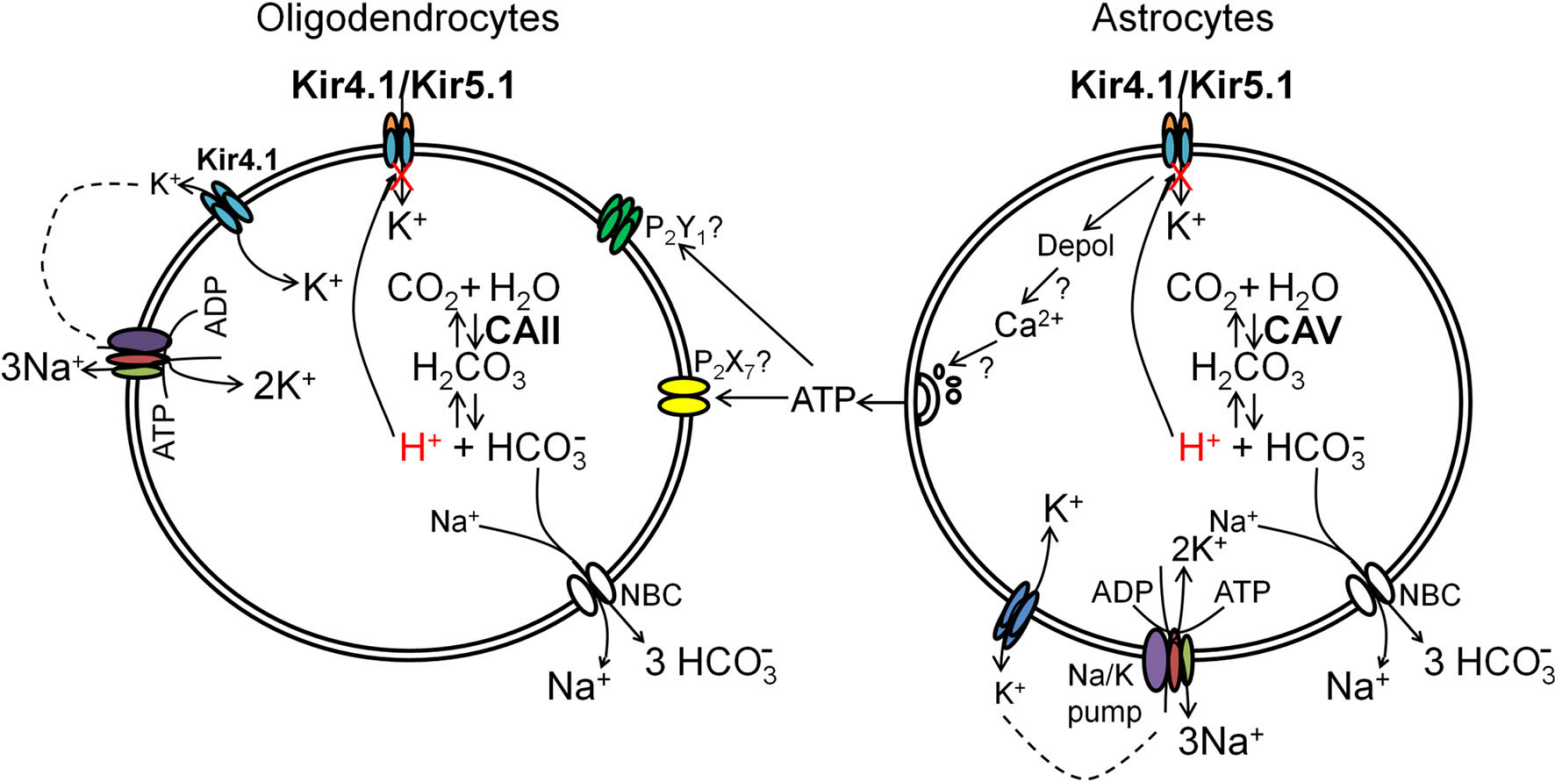
Functional implications of homomeric Kir4.1 and heteromeric Kir4.1/Kir5.1 channels in oligodendrocytes. Oligodendroglial expression of Kir4.1 channels indicates they may be important in uptake of excess K^+^ released during axonal action potential propagation, a function largely attribiuted to astrocytes. Due to their wrapping of axons, oligodendrocytes are exposed to large ionic and pH shifts during axonal electrical activity, and it is likely weakly rectifying homomeric Kir4.1 and strongly rectifying Kir4.1/Kir5.1 heteromeric channels are important in maintaining the negative resting membrane potential, which is essential for oligodendroglial and myelin integrity. Weakly rectifying homomeric Kir4.1 channels may preferentially extrude K^+^ and supply extracellular K^+^ for the Na^+^–K^+^-pumps, as described in transporting epithelia. In contrast, the pH sensitivity of heteromeric Kir4.1/Kir5.1 channels is likely to have a role in the CO_2_/pH chemosensation in glia, involving carbonic anhydrase that is enriched in astrocytes and oligodendrocytes. Furthermore, intracellular acidification and inhibition of Kir4.1/Kir5.1 channels has been shown to trigger release of ATP from astrocytes, which would act on oligodendroglial P2X and P2Y receptors to provide a mechanism of astrocyte–oligodendrocyte signaling in response to metabolic challenges, which has important implications for white matter physiology and pathology

Kir5.1 co-assembles with Kir4.1 to form functional pH-sensitive potassium channels (Yang et al. 2000; Cui et al. 2001; Pessia et al. 2001), which have been identified as astroglial CO_2_ chemoreceptors in the chemosensitive nuclei of the brainstem (Mulkey and Wenker 2011; Wu et al. 2004; Wenker et al. 2012). In white matter, oligodendrocytes are exposed to considerable ionic and pH shifts that take place during intense and continuous action potential propagation along axons (Kettenmann et al. 1990; Ransom 1992). This places a high metabolic load on oligodendrocytes and it is likely that Kir4.1/Kir5.1 channels in oligodendrocytes are involved in sensing changes in pH/CO_2_, which could be important in maintaining their RMP during metabolic stress (Fig. 8). As in other cells, a primary role of weakly rectifying homomeric Kir4.1 channels would be to supply Na^+^–K^+^ pumps, which is essential for K^+^ regulation by astrocytes and oligodendrocytes, whilst heteromeric Kir4.1/Kir5.1 channels would be essential for pH/CO_2_ sensation due to their unique pH sensitivity in the physiological range (Cui et al. 2001). Astrocytes express carbonic anhydrase (CA) V, while oligodendrocytes have CAII, which is critical for astroglial and oligodendroglial H^+^ regulation by the rapid conversion of CO_2_ and H_2_O to H_2_CO_3_, which dissociates to HCO_3_^−^ and H^+^ coupled to plasmalemmal Na^+^–H^+^-exchange and Na^+^-HCO_3_^−^ cotransport, as well as Na^+^-independent Cl^−^–HCO_3_^−^-exchange (Kettenmann et al. 1990; Boussouf and Gaillard 2000; Ro and Carson 2004). Kir4.1/Kir5.1 currents are regulated by CO_2_ and intracellular pH (Yang et al. 2000; Cui et al. 2001), hence inhibition of Kir4.1/Kir5.1 channels in response to intracellular acidification during intense axonal electrical activity and high metabolic demand would reduce inward K^+^ currents and help maintain the oligodendroglial membrane potential, which is essential for myelin integrity (Hawkins and Butt 2013). Furthermore, in the retrotrapezoid nucleus of the central chemoreceptors, inhibition of astroglial Kir4.1/Kir5.1 currents is the mechanism by which they sense an increase in extracellular CO_2_ and this triggers the release of ATP from astrocytes (Wenker et al. 2012). We have previously demonstrated that optic nerve astrocytes release ATP in response to axonal electrical activity (Hamilton et al. 2008, 2010) and astroglial ATP stimulates local blood flow (Butt 2011). Thus, astroglial Kir4.1/Kir5.1 channels provide a mechanism by which they sense increased H^+^/CO_2_ during axonal electrical activity and release ATP to increase local blood flow and maintain axonal function. In addition, ATP released by astrocytes would directly act on metabotropic ATP receptors (purinoceptors) on oligodendrocytes, namely P2Y_1_ and P2Y_12_ subtypes, which are prosurvival and maintain myelin integrity (Butt et al. 2014). However, in hypoxia/ischemia excessive release of ATP by astrocytes would activate oligodendroglial P2X_7_ receptors, which would cause myelin destruction (Matute et al. 2007), and blockade of Kir4.1/Kir5.1 may be a therapeutic target under these pathological conditions.

## References

[CR1] Azim K, Rivera A, Raineteau O, Butt AM (2014). GSK3beta regulates oligodendrogenesis in the dorsal microdomain of the subventricular zone via Wnt-beta-catenin signaling. Glia.

[CR2] Barlow AL, Macleod A, Noppen S, Sanderson J, Guerin CJ (2010). Colocalization analysis in fluorescence micrographs: verification of a more accurate calculation of Pearson’s correlation coefficient. Microsc Microanal.

[CR3] Bay V, Butt AM (2013). Relationship between glial potassium regulation and axon excitability: a role for glial Kir4.1 channels. Glia.

[CR4] Bhat RV, Axt KJ, Fosnaugh JS, Smith KJ, Johnson KA, Hill DE, Kinzler KW, Baraban JM (1996). Expression of the APC tumor suppressor protein in oligodendroglia. Glia.

[CR5] Bolton S, Butt AM (2006). Cyclic AMP-mediated regulation of the resting membrane potential in myelin-forming oligodendrocytes in the isolated intact rat optic nerve. Exp Neurol.

[CR6] Bolton S, Greenwood K, Hamilton N, Butt AM (2006). Regulation of the astrocyte resting membrane potential by cyclic AMP and protein kinase A. Glia.

[CR7] Boussouf A, Gaillard S (2000). Intracellular pH changes during oligodendrocyte differentiation in primary culture. J Neurosci Res.

[CR8] Brakeman JS, Gu SH, Wang XB, Dolin G, Baraban JM (1999). Neuronal localization of the adenomatous polyposis coli tumor suppressor protein. Neuroscience.

[CR9] Butt AM (2011). ATP: a ubiquitous gliotransmitter integrating neuron-glial networks. Semin Cell Dev Biol.

[CR10] Butt AM, Kalsi A (2006). Inwardly rectifying potassium channels (Kir) in central nervous system glia: a special role for Kir4.1 in glial functions. J Cell Mol Med.

[CR11] Butt AM, Pugh M, Hubbard P, James G (2004). Functions of optic nerve glia: axoglial signalling in physiology and pathology. Eye (London, England).

[CR12] Butt AM, Fern RF, Matute C (2014). Neurotransmitter signaling in white matter. Glia.

[CR13] Cui N, Giwa LR, Xu H, Rojas A, Abdulkadir L, Jiang C (2001). Modulation of the heteromeric Kir4.1–Kir5.1 channels by P(CO(2)) at physiological levels. J Cell Physiol.

[CR14] Derst C, Karschin C, Wischmeyer E, Hirsch JR, Preisig-Müller R, Rajan S, Engel H, Grzeschik K, Daut J, Karschin A (2001). Genetic and functional linkage of Kir5.1 and Kir2.1 channel subunits. FEBS Lett.

[CR15] Djukic B, Casper KB, Philpot BD, Chin L-S, McCarthy KD (2007). Conditional knock-out of Kir4.1 leads to glial membrane depolarization, inhibition of potassium and glutamate uptake, and enhanced short-term synaptic potentiation. J Neurosci.

[CR16] Fancy SP, Harrington EP, Baranzini SE, Silbereis JC, Shiow LR, Yuen TJ, Huang EJ, Lomvardas S, Rowitch DH (2014). Parallel states of pathological Wnt signaling in neonatal brain injury and colon cancer. Nat Neurosci.

[CR17] Fomina S, Howard TD, Sleator OK, Golovanova M, O’Ryan L, Leyland ML, Grossmann JGN, Collins RF, Prince SM (2011). Self-directed assembly and clustering of the cytoplasmic domains of inwardly rectifying Kir2.1 potassium channels on association with PSD-95. Biochim Biophys Acta.

[CR18] Fujita A, Horio Y, Higashi K, Mouri T, Hata F, Takeguchi N, Kurachi Y (2002). Specific localization of an inwardly rectifying K(+) channel, Kir4.1, at the apical membrane of rat gastric parietal cells; its possible involvement in K(+) recycling for the H(+)–K(+)-pump. J Physiol.

[CR19] Hamilton N, Vayro S, Kirchhoff F, Verkhratsky A, Robbins J, Gorecki DC, Butt AM (2008). Mechanisms of ATP- and glutamate-mediated calcium signaling in white matter astrocytes. Glia.

[CR20] Hamilton N, Vayro S, Wigley R, Butt AM (2010). Axons and astrocytes release ATP and glutamate to evoke calcium signals in NG2-glia. Glia.

[CR21] Hawkins V, Butt A (2013). TASK-1 channels in oligodendrocytes: a role in ischemia mediated disruption. Neurobiol Dis.

[CR22] Hibino H, Fujita A, Iwai K, Yamada M, Kurachi Y (2004). Differential assembly of inwardly rectifying K+ channel subunits, Kir4.1 and Kir5.1, in brain astrocytes. J Biol Chem.

[CR23] Hibino H, Inanobe A, Furutani K, Murakami S, Findlay I, Kurachi Y (2010). Inwardly rectifying potassium channels: their structure, function, and physiological roles. Physiol Rev.

[CR24] Hirrlinger PG, Scheller A, Braun C, Quintela-Schneider M, Fuss B, Hirrlinger J, Kirchhoff F (2005). Expression of reef coral fluorescent proteins in the central nervous system of transgenic mice. Mol Cell Neurosci.

[CR25] Horio Y (2001). Potassium channels of glial cells: distribution and function. Jpn J Pharmacol.

[CR26] Horio Y, Hibino H, Inanobe A, Yamada M, Ishii M, Tada Y, Satoh E, Hata Y, Takai Y, Kurachi Y (1997). Clustering and enhanced activity of an inwardly rectifying potassium channel, Kir4.1, by an anchoring protein, PSD-95/SAP90. J Biol Chem.

[CR27] Howe MW, Feig SL, Osting SMK, Haberly LB (2008). Cellular and subcellular localization of Kir2.1 subunits in neurons and glia in piriform cortex with implications for K+ spatial buffering. J Comp Neurol.

[CR28] Ishii M, Fujita A, Iwai K, Kusaka S, Higashi K, Inanobe A, Hibino H, Kurachi Y (2003). Differential expression and distribution of Kir5.1 and Kir4.1 inwardly rectifying K+ channels in retina. Am J Physiol Cell Physiol.

[CR29] Kalsi AS, Greenwood K, Wilkin G, Butt AM (2004). Kir4.1 expression by astrocytes and oligodendrocytes in CNS white matter: a developmental study in the rat optic nerve. J Anat.

[CR30] Kang SJ, Cho S-H, Park K, Yi J, Yoo SJ, Shin KS (2008). Expression of Kir2.1 channels in astrocytes under pathophysiological conditions. Mol Cells.

[CR31] Kaufhold M-A, Krabbenhöft A, Song P, Engelhardt R, Riederer B, Fährmann M, Klöcker N, Beil W, Manns M, Hagen SJ, Seidler U (2008). Localization, trafficking, and significance for acid secretion of parietal cell Kir4.1 and KCNQ1 K+ channels. Gastroenterology.

[CR32] Kettenmann H, Ransom BR, Schlue WR (1990). Intracellular pH shifts capable of uncoupling cultured oligodendrocytes are seen only in low HCO_3_− solution. Glia.

[CR33] Kofuji P, Newman EA (2004). Potassium buffering in the central nervous system. Neuroscience.

[CR34] Kofuji P, Ceelen P, Zahs KR, Surbeck LW, Lester HA, Newman EA (2000). Genetic inactivation of an inwardly rectifying potassium channel (Kir4.1 subunit) in mice: phenotypic impact in retina. J Neurosci.

[CR35] Kofuji P, Biedermann B, Siddharthan V, Raap M, Iandiev I, Milenkovic I, Thomzig A, Veh RdW, Bringmann A, Reichenbach A (2002). Kir potassium channel subunit expression in retinal glial cells: implications for spatial potassium buffering. Glia.

[CR36] Konstas A-A, Korbmacher C, Tucker SJ (2003). Identification of domains that control the heteromeric assembly of Kir5.1/Kir4.0 potassium channels. Am J Physiol Cell Physiol.

[CR37] Lachheb S, Fo Cluzeaud, Bens M, Genete M, Hibino H, Sp Lourdel, Kurachi Y, Vandewalle A, Teulon J, Paulais M (2008). Kir4.1/Kir5.1 channel forms the major K+ channel in the basolateral membrane of mouse renal collecting duct principal cells. Am J Physiol Renal Physiol.

[CR38] Lang J, Maeda Y, Bannerman P, Xu J, Horiuchi M, Pleasure D, Guo F (2013). Adenomatous polyposis coli regulates oligodendroglial development. J Neurosci.

[CR39] Leonoudakis D, Mailliard W, Wingerd K, Clegg D, Vandenberg C (2001). Inward rectifier potassium channel Kir2.2 is associated with synapse-associated protein SAP97. J Cell Sci.

[CR40] Matute C, Torre I, Perez-Cerda F, Perez-Samartin A, Alberdi E, Etxebarria E, Arranz AM, Ravid R, Rodriguez-Antiguedad A, Sanchez-Gomez M, Domercq M (2007). P2X(7) receptor blockade prevents ATP excitotoxicity in oligodendrocytes and ameliorates experimental autoimmune encephalomyelitis. J Neurosci.

[CR41] Menichella DM, Majdan M, Awatramani R, Goodenough DA, Sirkowski E, Scherer SS, Paul DL (2006). Genetic and physiological evidence that oligodendrocyte gap junctions contribute to spatial buffering of potassium released during neuronal activity. J Neurosci.

[CR42] Mulkey DK, Wenker IC (2011). Astrocyte chemoreceptors: mechanisms of H+ sensing by astrocytes in the retrotrapezoid nucleus and their possible contribution to respiratory drive. Exp Physiol.

[CR43] Nehring RB, Wischmeyer E, Döring F, Veh RW, Sheng M, Karschin A (2000). Neuronal inwardly rectifying K(+) channels differentially couple to PDZ proteins of the PSD-95/SAP90 family. J Neurosci.

[CR44] Neusch C, Rozengurt N, Jacobs RE, Lester HA, Kofuji P (2001). Kir4.1 potassium channel subunit is crucial for oligodendrocyte development and in vivo myelination. J Neurosci.

[CR45] Neusch C, Papadopoulos N, Müller M, Maletzki I, Winter SM, Hirrlinger J, Handschuh M, Bähr M, Richter DW, Kirchhoff F, Hüsmann S (2006). Lack of the Kir4.1 channel subunit abolishes K+ buffering properties of astrocytes in the ventral respiratory group: impact on extracellular K+ regulation. J Neurophysiol.

[CR46] Nolte C, Matyash M, Pivneva T, Schipke CG, Ohlemeyer C, Hanisch UK, Kirchhoff F, Kettenmann H (2001). GFAP promoter-controlled EGFP-expressing transgenic mice: a tool to visualize astrocytes and astrogliosis in living brain tissue. Glia.

[CR47] Olsen ML, Sontheimer H (2008). Functional implications for Kir4.1 channels in glial biology: from K+ buffering to cell differentiation. J Neurochem.

[CR48] Paulais M, Bloch-Faure M, Picard N, Jacques T, Ramakrishnan SK, Keck M, Sohet F, Eladari D, Houillier P, Sp Lourdel, Teulon J, Tucker SJ (2011). Renal phenotype in mice lacking the Kir5.1 (Kcnj16) K+ channel subunit contrasts with that observed in SeSAME/EAST syndrome. Proc Natl Acad Sci USA.

[CR49] Pegan S, Tan J, Huang A, Slesinger PA, Riek R, Choe S (2007). NMR studies of interactions between C-terminal tail of Kir2.1 channel and PDZ1, 2 domains of PSD95. Biochemistry.

[CR50] Pessia M, Imbrici P, D’Adamo MC, Salvatore L, Tucker SJ (2001). Differential pH sensitivity of Kir4.1 and Kir4.2 potassium channels and their modulation by heteropolymerisation with Kir5.1. J Physiol.

[CR51] Poopalasundaram S, Knott C, Shamotienko OG, Foran PG, Dolly JO, Ghiani CA, Gallo V, Wilkin GP (2000). Glial heterogeneity in expression of the inwardly rectifying K(+) channel, Kir4.1, in adult rat CNS. Glia.

[CR52] Ransom BR (1992). Glial modulation of neural excitability mediated by extracellular pH: a hypothesis. Prog Brain Res.

[CR53] Rapedius M, Paynter JJ, Fowler PW, Shang L, Sansom MSP, Tucker SJ, Baukrowitz T (2007). Control of pH and PIP2 gating in heteromeric Kir4.1/Kir5.1 channels by H-bonding at the helix-bundle crossing. Channels (Austin, Tex).

[CR54] Ro HA, Carson JH (2004). pH microdomains in oligodendrocytes. J Biol Chem.

[CR55] Rojas A, Cui N, Su J, Yang L, Muhumuza J-P, Jiang C (2007). Protein kinase C dependent inhibition of the heteromeric Kir4.1–Kir5.1 channel. Biochim Biophys Acta.

[CR56] Rojas A, Su J, Yang L, Lee M, Cui N, Zhang X, Fountain D, Jiang C (2008). Modulation of the heteromeric Kir4.1–Kir5.1 channel by multiple neurotransmitters via Galphaq-coupled receptors. J Cell Physiol.

[CR57] Seifert G, Hüttmann K, Binder DK, Hartmann C, Wyczynski A, Neusch C, Steinhäuser C (2009). Analysis of astroglial K+ channel expression in the developing hippocampus reveals a predominant role of the Kir4.1 subunit. J Neurosci.

[CR58] Stolt CC, Schlierf A, Lommes P, Hillgartner S, Werner T, Kosian T, Sock E, Kessaris N, Richardson WD, Lefebvre V, Wegner M (2006). SoxD proteins influence multiple stages of oligodendrocyte development and modulate SoxE protein function. Dev Cell.

[CR59] Tanemoto M, Kittaka N, Inanobe A, Kurachi Y (2000). In vivo formation of a proton-sensitive K+ channel by heteromeric subunit assembly of Kir5.1 with Kir4.1. J Physiol.

[CR60] Tanemoto M, Fujita A, Higashi K, Kurachi Y (2002). PSD-95 mediates formation of a functional homomeric Kir5.1 channel in the brain. Neuron.

[CR61] Tanemoto M, Abe T, Onogawa T, Ito S (2004). PDZ binding motif-dependent localization of K+ channel on the basolateral side in distal tubules. Am J Physiol Renal Physiol.

[CR62] Tanemoto M, Toyohara T, Abe T, Ito S (2008). MAGI-1a functions as a scaffolding protein for the distal renal tubular basolateral K+ channels. J Biol Chem.

[CR63] Tang X, Taniguchi K, Kofuji P (2009). Heterogeneity of Kir4.1 channel expression in glia revealed by mouse transgenesis. Glia.

[CR64] Tucker SJ, Imbrici P, Salvatore L, D’Adamo MC, Pessia M (2000). pH dependence of the inwardly rectifying potassium channel, Kir5.1, and localization in renal tubular epithelia. J Biol Chem.

[CR65] Ulbricht E, Pannicke T, Hollborn M, Raap M, Goczalik I, Iandiev I, Härtig W, Uhlmann S, Wiedemann P, Reichenbach A, Bringmann A, Francke M (2008). Proliferative gliosis causes mislocation and inactivation of inwardly rectifying K(+) (Kir) channels in rabbit retinal glial cells. Exp Eye Res.

[CR66] Verkhratsky A, Steinhäuser C (2000). Ion channels in glial cells. Brain Res Brain Res Rev.

[CR67] Wenker IC, Kréneisz O, Nishiyama A, Mulkey DK (2012). Astrocytes in the retrotrapezoid nucleus sense H+ by inhibition of a Kir4.1–Kir5.1-like current and may contribute to chemoreception by a purinergic mechanism. J Neurophysiol.

[CR68] Wu J, Xu H, Shen W, Jiang C (2004). Expression and coexpression of CO_2_-sensitive Kir channels in brainstem neurons of rats. J Membr Biol.

[CR69] Xu H, Cui N, Yang Z, Qu Z, Jiang C (2000). Modulation of kir4.1 and kir5.1 by hypercapnia and intracellular acidosis. J Physiol.

[CR100] Yamamoto Y, Ishikawa R, Omoe K, Yoshikawa N, Yamaguchi-Yamada M, Taniguchi K (2008). Immunohistochemical distribution of inwardly rectifying K+ channels in the medulla oblongata of the rat. J Vet Med Sci.

[CR70] Yang Z, Xu H, Cui N, Qu Z, Chanchevalap S, Shen W, Jiang C (2000). Biophysical and molecular mechanisms underlying the modulation of heteromeric Kir4.1–Kir5.1 channels by CO_2_ and pH. J Gen Physiol.

